# Interplay of the 3D genome and epigenome in androgen-driven prostate cancer

**DOI:** 10.1080/17501911.2026.2676129

**Published:** 2026-05-22

**Authors:** Elyssa M. Campbell, Dayna Challis, Susan J. Clark, Joanna Achinger-Kawecka

**Affiliations:** a Cancer Ecosystems Program, Garvan Institute of Medical Research, Sydney, NSW, Australia; b School of Clinical Medicine, UNSW Medicine and Health, Sydney, NSW, Australia; c South Australian ImmunoGENomics Cancer Institute (SAiGENCI), College of Health, Adelaide University, Adelaide, SA, Australia

**Keywords:** Chromatin, prostate cancer, androgen receptor, epigenetics, 3D genome

## Abstract

Understanding of the therapeutic landscape for androgen-driven prostate cancer has undergone significant evolution, yet treatment resistance remains an ongoing challenge. The androgen receptor (AR) plays a central role in androgen-dependent prostate tumor growth, and therapeutic agents targeting AR represent the main treatment modality for prostate cancer. AR transcriptional activity is tightly regulated by coregulatory transcription factors and their DNA binding and activity. However, the precise interactions between AR and three-dimensional (3D) chromatin structure and its dynamic remodeling represent a critical yet underexploited therapeutic frontier. Here, we present a perspective on how the spatial architecture of the epigenome, including 3D chromatin structure and dynamics, orchestrates dysregulation of transcription factor networks that help to drive disease progression in androgen-driven prostate cancer. We argue that successful therapeutic intervention requires moving beyond linear epigenetic marks toward a three-dimensional understanding of the epigenetic landscape. Such a shift is essential to capture the complex enhancer-promoter communications and chromatin state transitions that define the disease. Drawing on recent advances in prostate cancer biology and technological developments, we propose a unique integrated framework centered on patient-specific 3D epigenomic vulnerabilities for next-generation epigenetic therapies designed to disrupt oncogenic transcriptional programs by specifically targeting chromatin topology.

## Plain Language Summary

Prostate cancer is commonly driven by hormones called androgens. Most current treatments work by blocking the androgen receptor (AR), which is a protein that acts like a master switch to turn on genes that help the cancer grow. While these treatments can be effective, many patients eventually develop resistance and relapse. Traditionally, scientists have studied DNA as a linear “instruction manual.” In this review, we argue that we need to look at the bigger picture: the three-dimensional (3D) shape of DNA within the cell and the changes to the epigenetic marks on the DNA and the proteins that cover the DNA. DNA inside the cell is folded into complex loops and structures that bring distant “control switches” (enhancers) into contact with specific genes (promoters). In prostate cancer, this 3D folding gets “rewired,” allowing the cancer to bypass standard treatments and keep growing. We propose a new approach to improving treatment outcomes that focuses on mapping this 3D architecture and epigenetic marks in individual patient tumors. This can be achieved through combining advanced genomic tools to study this complex 3D DNA organization with new types of drugs that target these DNA structures, to find more effective and longer-lasting therapies when transitional hormone therapies fail.

## 1. Introduction

Prostate cancer (PCa) represents a model for understanding how epigenetic driven plasticity enables therapeutic resistance. While androgen deprivation therapy initially achieves remarkable clinical responses, progression to castration-resistant prostate cancer (CRPC) and subsequently to neuroendocrine prostate cancer (NEPC) highlights the adaptive capacity of the prostate cancer epigenomes. The androgen receptor (AR), a nuclear receptor that binds DNA at androgen response elements (AREs), orchestrates pro-tumorigenic transcriptional programs essential for disease progression [1].

Current therapeutic strategies have focused predominantly on disrupting AR ligand binding with agents such as enzalutamide. While effective in early-stage disease, these approaches become ineffective as tumors evolve mechanisms to circumvent androgen-targeted therapies, including AR amplification, splice variants, and gene fusions [2]. Critically, the progression from localized disease to advanced CRPC involves comprehensively reprogramming the genome-wide collection of AR binding sites, referred to as the AR cistrome [3]. This cistrome reprogramming is not merely a consequence of genetic alterations but occurs through widespread epigenetic remodeling at the AREs.

An extreme example of epigenome reprogramming is the emergence of neuroendocrine-like (NE) states following androgen receptor pathway inhibitor (ARPI) treatment. While NEPC accounts for less than 2% of newly diagnosed cases, up to 17% of CRPC tumors evolve this phenotype following prolonged androgen deprivation and ARPI treatment [4,5]. This transition involves transcriptional lineage switching driven by AR loss or very low AR expression, with subsequent tumor growth proceeding through AR-independent mechanisms [6,7]. The lineage switch to NEPC involves dysregulation of epigenetic modifiers, including the histone methyltransferase EZH2 and DNA methyltransferase DNMT1, which cooperate with lineage-determining transcription factors (TFs) to establish distinct patterns of chromatin accessibility that can repress the AR-dependent transcriptional program [8].

Critically, recent work shows that this epigenetic reprogramming is accompanied by reorganization of three-dimensional (3D) chromatin architecture, where pioneer factor FOXA2 binds at NE-specific enhancers to induce expression of the neural transcription factor NKX2-1, which in turn interacts with enhancer-bound FOXA2 through chromatin looping to drive luminal-to-NE transformation [9]. The underlying molecular basis of this resistance mechanism remains incompletely understood, but a suite of epigenetic mechanisms appear to play a central role in establishing, maintaining, and adapting cell-lineage transcriptional programs [6,10–13].

Emerging evidence demonstrates that DNA methylation, chromatin accessibility, nucleosome positioning, and particularly 3D chromatin interactions represent important layers of epigenetic control that can be targeted for cancer therapy. Importantly, these layers do not function independently, but rather in convergence at key regulatory elements that establish and maintain the oncogenic transcriptional programs driving disease progression and treatment resistance. In this Perspective, we argue that the future of epigenetic cancer therapy lies in targeting the specific features of chromatin that enable oncogenic TF networks to function, with 3D chromatin organization representing the most therapeutically underexploited of these layers and the one most capable of integrating the other layers of the epigenome landscape into an actionable framework. We focus on prostate cancer as an illustrative model, but the principles discussed extend broadly to other hormone-driven and TF-dependent malignancies.

### 1.1. Chromatin landscape in androgen-dependent prostate cancer

**Figure 1.**
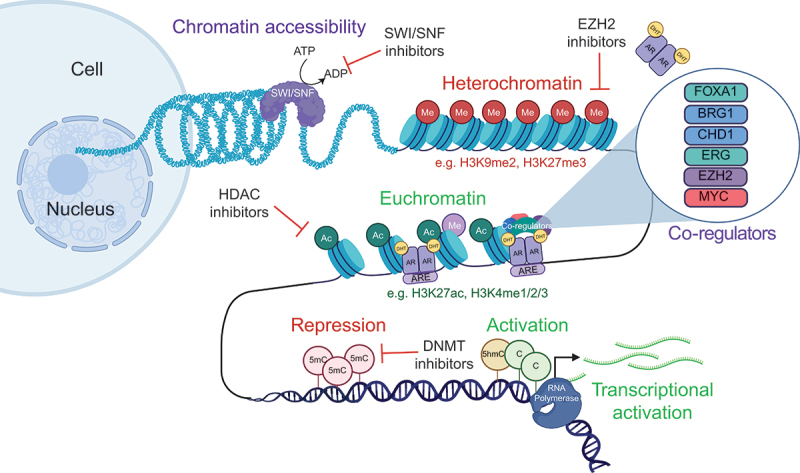
Chromatin landscape of androgen-driven transcription. Chromatin accessibility is regulated by chromatin remodeling complexes such as SWI/SNF. Heterochromatin (closed chromatin) is marked by repressive histone modifications (methylation – H3K9me2, H3K27me3) and euchromatin (open chromatin) is marked by active histone modifications (H3K27ac and H3K4me1/2/3). These chromatin states are associated with transcription factor activity on the chromatin and their interactions with co-regulators. In the case of androgen-driven prostate cancer, upon hormone (DHT) stimulation, AR and its co-regulators bind to DNA to initiate chromatin remodeling and AR-mediated transcription. Epigenetic inhibitors are being tested in the context of AR-driven prostate cancer to target these chromatin states. SWI/SNF inhibitors are used to target chromatin remodeling, EZH2 inhibitors are used to target histone repressive marks in heterochromatic domains, HDAC inhibitors are used to target histone deacetylation in euchromatic domains and DNMT inhibitors are used to target DNA methylation. Created in BioRender. Achinger-Kawecka, J. (2026) https://BioRender.com/iauabhr.

Abbreviations: AR: Androgen receptor, Me: Methylation, Ac: Acetylation, ARE: Androgen response elements, DHT: Dihydrotestosterone, DNMT: DNA methyltransferase, EZH2: Enhancer of zeste homolog 2, HDAC: histone deacetylase, SWI/SNF: Switch/Sucrose Non-fermenting complex.

The chromatin landscape, encompassing 3D chromatin structure, chromatin accessibility and histone modifications, represent overarching epigenetic regulatory layers that together facilitate transcription factor binding and spatiotemporal gene expression (Figure 1) [14]. However, viewing these epigenetic features as linear ‘marks’ on the chromatin is oversimplifying the complexity of AR driven gene regulation. Chromatin is instead organized into a complex and highly coordinated three-dimensional structure. At the largest scale, chromosomes occupy distinct nuclear territories [15]. Within these territories, the genome is partitioned into transcriptionally active (A) and inactive (B) compartments, broadly corresponding to euchromatin and heterochromatin, respectively [15]. These compartments are further subdivided into topologically associating domains (TADs), which represent regions of enriched local chromatin interactions that constrain regulatory contacts [16]. At the finest scale, chromatin loops form within TADs to bring distal regulatory elements, such as enhancers and promoters together, providing the most direct mechanism for fine-tuning gene expression [17–21].

In PCa, this 3D chromatin organization is extensively reprogrammed within the AR cistrome during PCa development and progression [22,23]. For instance, pioneer transcription factors FOXA1 and HOXB13 direct AR to distinct genomic regions. This dependency shifts in castrate-resistant disease where the AR splice variant AR-V7 exhibits selective dependence on HOXB13 rather than FOXA1 for chromatin accessibility [24]. Notably, AR binding occurs predominantly at intergenic regions containing cis-regulatory enhancer and promoter elements [6,25–28]. At these sites, AR occupancy induces rapid transcriptional changes, by co-binding with co-regulatory TFs, driving the formation of 3D chromatin interactions between AR-bound enhancers and target gene promoters [29–31].

Recent work has added to our understanding of how AR activation temporally coordinates epigenetic changes and 3D chromatin reorganization to drive downstream gene transcription in CRPC cells. Rather than substantially rewiring chromatin loops AR binding increases the contact frequency of preexisting loops between AR-bound enhancers and their target promoters [30,31]. These androgen-induced changes are both rapid and highly transient. AR-induced 3D chromatin interactions that are rapidly gained within 30 minutes are subsequently resolved within a few hours [30,31]. Additionally, these studies show that not all AR-bound loops are equal; there are dominant enhancer loops within multi-enhancer networks which disproportionately impact this gene expression [31]. The transient nature of these interactions suggests that the window of therapeutic vulnerability to androgen inhibition is narrow and temporally defined. Furthermore, the identification of dominant enhancer loops as key drivers of androgen-mediated transcription could provide more specific therapeutic targets, reducing the off-target effects. These novel insights into AR-driven mechanisms make clearer the complexity of this temporally coordinated regulatory system in PCa and the vulnerabilities which could be exploited with targeted therapies.

3D chromatin reprogramming extends beyond rapid loop dynamics to fundamental changes in cell identity and large-scale chromatin organization. For instance, 3D chromatin reorganization is also critical in lineage switching, where NE transformation is characterized by distinct 3D chromatin interactions at FOXA2 binding to NE enhancers. These interactions, in turn, mediate local DNA demethylation and transcription of the neural transcription factor *NKX2-1* [9]. On a broader scale, 3D chromatin reprogramming is also evident at the higher-order level, where analysis of A/B compartmentalization patterns stratifies prostate cancer patients into low de-compartmentalization (LDD) and high de-compartmentalization (HDD) subtypes, each with distinct transcriptional programs and clinical outcomes with predictive prognostic value [32]. Finally, integrated analyses of 3D chromatin structure and epigenome remodeling in mCRPC biopsy samples revealed interactions between 3D genome and genetic variation, including AR gene amplifications in extrachromosomal DNA (ecDNA) [33]. AR gene is frequently co-amplified in ecDNAs with LINE1 elements, which provide binding sites for its co-activator factors, FOXA1 and HOXB13, and establish new 3D chromatin interactions driving AR overexpression [34].

Another important aspect of the 3D chromatin landscape is chromatin accessibility that can dictate where TFs bind. Chromatin accessibility is controlled by ATP-dependent chromatin remodeling complexes that mobilize nucleosomes. These complexes comprise two functional subgroups with subunits exhibiting preferential binding at regions marked by either active or repressive histone modifications [35]. Both groups include subunits from the SWI/SNF, INO80, ISWI, and CHD families that interact with chromatin and DNA to acetylate, methylate, phosphorylate, and ubiquitinate histones, or mediate nucleosome repositioning [36]. Several identified AR cofactors, such as FOXA1, GATA2, ERG and HOXB13 [37] possess intrinsic chromatin remodeling capabilities or recruit chromatin-modifying enzymes [38] to facilitate AR binding. These cofactors can function as pioneer factors that engage compacted chromatin prior to AR binding, displacing nucleosomes and creating an accessible chromatin state. The activity of these pioneer factors is frequently disrupted across disease stages, resulting in reprogramed chromatin accessibility landscape [6,37,39].

Profiling the chromatin accessibility landscape has been crucial for classifying the disease. For example, accessibility profiling in a diverse biobank of organoids and patient-derived xenografts (PDXs) characterized novel metastatic CRPC (mCRPC) subtypes and identified master TFs driving AR-negative CRPCs [40]. A recent first-in-field ATAC-seq study conducted in the largest cohort (*n* = 70) of mCRPC tissue biopsies identified changes in chromatin accessibility around regulatory sites that drive transcriptional regulation associated with different mCRPC subtypes [41]. Additional studies have utilized chromatin accessibility and multi-omic analyses to identify a novel role for transposable elements in activating the AR locus itself, as well as AR and co-factor transcriptional activity in mCRPC [42]. Specifically at the AR gene locus, clusters of accessible LINE1 elements are co-amplified with the AR gene. These amplifications dramatically increase AR cofactor binding sites and 3D chromatin loops, creating a self-reinforcing positive feedback loop associated with extreme AR overexpression in these mCRPC patients.

Collectively, these findings establish that 3D chromatin reprogramming in prostate cancer governs AR-driven transcriptional programs across multiple spatial and temporal scales. These range from transient enhancer-promoter loops and lineage-defining chromatin reorganization to compartment-level reprogramming, ecDNA-mediated structural variation, and nucleosome remodeling-dependent chromatin accessibility. Each represents a distinct layer of therapeutic vulnerability that cannot be captured by linear epigenetic profiling alone.

### 1.2. The transcription factor networks driving androgen-dependent oncogenesis

As prostate cancer advances from hormone-sensitive to castration-resistant states, distinct AR signaling profiles emerge, characterized by coordinated transcription factor co-regulator binding and epigenetic reprogramming that correlate with disease stage [26,43,44]. Cistrome profiling through mapping AR occupancy in normal, primary, and metastatic PCa samples has demonstrated that AR binding exhibits fundamentally different genomic distributions [45,46]. In CRPC, AR binding re-localizes to sites driving oncogene expression, including MYC, and these binding patterns predict disease progression and patient outcomes [45].

Additionally, resistance to AR-targeted therapy can also arise through specific regulatory circuits that sustain AR signaling or activate alternative transcriptional programs independently of androgen. For example, the lnc-OPHN1-5/AR/hnRNPA1 complex has been shown to sustain AR signaling and modulate enzalutamide sensitivity, while AR-Beclin 1 complex-modulated growth factor signaling has been implicated in enzalutamide resistance [47,48]. These resistance mechanisms underscore the importance of combination strategies that simultaneously target both the chromatin topology sustaining oncogenic TF networks and the alternative signaling axes through which resistance emerges.

Recent work has revealed that AR and H3K27ac chromatin binding sites can stratify localized hormone-sensitive disease from metastatic CRPC [43,49]. In malignant states, AR repositions to genomic loci characterized by low DNA methylation and accessible chromatin, which were pre-marked by pioneer factors FOXA1 and HOXB13. Notably, these pre-marked sites show greater enrichment in localized disease compared to mCRPC, suggesting progressive chromatin remodeling accompanies disease evolution. This shift implies that as disease advances, the pioneer factor landscape itself is remodeled, with FOXA1 and HOXB13 pre-marking enabling altered AR binding cistrome, contributing directly to the emergence of castration resistance. Further, state-specific AR binding patterns associate with distinct gene expression signatures and clinical outcomes, underscoring the functional relevance of cistromic reorganization [26]. This reprogramming is not driven by AR alone but by a network of interacting TFs, including key factors FOXA1, ERG, HOXB13 and MYC (summarized in Table 1). Understanding the temporal sequence by which occupancy of these pioneer factors and co-regulators is redirected during disease progression represents a critical mechanistic gap with direct implications for identifying early epigenetic vulnerabilities that could be targeted before the transition to a castration-resistant state.

**Table 1.** Summary of key transcription factors, their associated chromatin features, and their functional consequences in androgen-driven prostate cancer.

Transcription factor	Chromatin role	Epigenome / 3D genome features	Functional consequences
AR	Master transcription factor	Binds distal enhancers; increases contact frequency of preexisting enhancer – promoter loops; associated with H3K27ac and cofactor recruitment (FOXA1, HOXB13, BRD4)	Drives lineage-specific transcription; coordinates rapid, transient gene activation; reprogrammed in advanced disease
FOXA1	Pioneer factor	Establishes enhancer accessibility (H3K4me1/2); facilitates AR binding; cooperates with BRG1 and CHD1	Defines AR cistrome; regulates lineage-specific transcription; promotes AR redistribution and lineage plasticity
HOXB13	Lineage transcription factor	Directs AR binding to alternative enhancer regions; co-occupies AR enhancers; supports AR binding independent of FOXA1	Reprograms AR cistrome; sustains AR signaling in CRPC; promotes lineage plasticity
ERG	ETS transcription factor	Reprograms AR cistrome; recruits epigenetic modifiers (e.g., EZH2, HDACs); alters enhancer – promoter loops; cooperates with SWI/SNF	Represses AR-driven transcription; induces bivalent chromatin; promotes dedifferentiation and oncogenic programs
MYC	Transcriptional amplifier	Co-binds AR at enhancers/super-enhancers; associated with H3K27ac; modulates enhancer – promoter looping; interacts with BRD4 and EZH2	Amplifies oncogenic transcription; antagonizes AR signaling; promotes dedifferentiation and lineage plasticity

Abbreviations: AR: Androgen receptor, CRPC: Castration-resistant prostate cancer, EZH2: Enhancer of zeste homolog 2, HDAC: histone deacetylase, SWI/SNF: Switch/Sucrose Non-fermenting complex.

### 1.3. The pioneer factor FOXA1

FOXA1 (forkhead box A1) has emerged as a critical mediator of chromatin accessibility in prostate cancer. The *FOXA1* gene itself is frequently mutated in primary and metastatic prostate tumors and several key non-coding mutations have been identify at its locus and cistrome [50,51]. Originally identified for its role in initiating chromatin opening during embryonic development [52], FOXA1 functions as a pioneer factor in nuclear receptor signaling pathways, including those mediated by AR [46,53,54]. FOXA1 interacts directly with AR through its hinge domain, creating cell-lineage-specific chromatin remodeling patterns [53,55]. This interaction underlies the role of FOXA1 in lineage-specific AR signaling and its contribution to lineage switching as AR binding is rerouted during disease progression and neuroendocrine transition [39].

The pioneer function of FOXA1 depends on histone methylation states, particularly enhancer-associated H3K4me1 and H3K4me2 marks deposited by MLL2/3 complexes at cell-type-specific FOXA1 sites [53]. Clinically, FOXA1 overexpression frequently associates with poor outcomes in PCa patients, while low FOXA1 expression correlates with a favorable prognosis irrespective of AR expression [56,57]. In contrast, FOXA1 overexpression is associated with good clinical outcomes in hormone-sensitive breast cancer patients, which highlights the need for understanding FOXA1-directed cistromes in more depth [56].

Several studies have demonstrated that FOXA1 orchestrates chromatin remodeling through recruitment of ATP-dependent remodeling complexes in a chromatin state-dependent manner. FOXA1-pioneered AR binding requires the SWI/SNF subunit BRG1 (encoded by the *SMARCA4* gene), which pre-marks promoters of genes driving PCa proliferation [58]. These BRG1-bound sites are marked by active, accessible chromatin where FOXA1 modulates hormone-driven responses [59]. Additionally, chromodomain helicase DNA-binding protein 1 (CHD1) mediates AR sensitivity in mCRPC by regulating AR and cofactor occupancy at prostate-specific enhancers, including FOXA1 binding sites [60]. CHD1 restricts the AR cistrome to maintain normal prostate transcription, enabling a permissive state for coordinated FOXA1 and AR binding [60]. Consequently, CHD1 loss represents a key transition point toward aggressive mCRPC and NEPC states [61–63].

Together, these findings position FOXA1 as a master regulator of the AR cistrome. FOXA1 pioneer activity and chromatin remodeling dependencies make it the central node linking chromatin state to transcriptional output across a spectrum of prostate cancer progression phenotypes.

### 1.4. ERG and ETS factor dysfunction

While FOXA1 acts as a pioneer to open the chromatin landscape, other transcription factors, for example ERG, function to hijack and rewrite it. ERG, a member of the E26 transformation-specific (ETS) TF family, plays a critical cooperative role within AR transcriptional networks. ETS factors bind GGA(A/T) DNA motifs and participate in cell-cycle, apoptosis, and differentiation signaling cascades [64]. Gene fusions involving ETS factors are among the most common genetic alterations in PCa, with ERG overexpression found in the majority of cases, 50% of these due to TMPRSS2-ERG fusion [65,66]. As TMPRSS2 is androgen-responsive and constitutively expressed in prostate tissue, this fusion results in high expression of truncated ERG that disrupts androgen signaling by inhibiting AR expression and competing for AR binding sites [67].

ERG’s ability to induce expression of the AR-target gene *KLK3*, encoding PSA, exemplifies its role in distorting the AR cistrome [68]. Mechanistically, ERG represses AR-driven transcription by directly recruiting the H3K27 methyltransferase EZH2 (Enhancer of Zeste Homolog 2), the catalytic subunit of Polycomb Repressive Complex 2 (PRC2), to ERG-bound AREs, resulting in H3K27me3 deposition and transcriptional silencing [67]. A subset of promoters becomes simultaneously marked by repressive H3K27me3 and active H3K4me3 modifications, and these bivalent promoters regulate developmental lineage-specific genes [67], suggesting a role of ERG in cellular dedifferentiation that may underlie mCRPC and NEPC development.

While ERG does not directly remodel chromatin independently, its overexpression triggers massive chromatin reorganization through interactions with various epigenetic modifiers, including p300, HDAC1, HDAC2, EZH2, KDM4A, PRMT5, and SETDB1 [67,69–76]. Androgen-induced interaction hubs, where ERG colocalises with AR, at one or both ends of 3D chromatin enhancer-promoter loops, exhibit bidirectional transcription of PCa-associated protein-coding genes and long non-coding RNAs [29]. These ERG-AR interaction hubs can be further stratified by their cofactor composition: loops anchored by both AR and ERG enrich for FOXA1, EZH2, and HDAC3; ERG-only anchors enrich for HDAC1 and BRD2/3/4; AR-only anchors enrich for FOXA1, RNA Polymerase II, HDAC2/3, and GAPBPA [29]. Each configuration supports a model whereby ERG represses AR-activated transcription through epigenetic modification of ARE regions. Critically, this cofactor stratification implies that distinct 3D loop configurations are not functionally equivalent. Dual ERG-AR anchored loops, enriched for EZH2 and HDAC3, are likely to enforce a more stably repressed chromatin state at AR target loci, whereas ERG-only anchors, enriched for BRD2/3/4, may maintain a poised enhancer configuration permissive to transcriptional activation, suggesting that the therapeutic impact of targeting any single epigenetic modifier will depend on which ERG-loop configuration is dominant in the tumor.

Finally, TMPRSS2-ERG overexpression induces chromatin remodeling by binding SWI/SNF subunits, including BRG1, driving prostate oncogenesis and lineage transitions [71]. ERG redirects SWI/SNF (BAF) ATP-dependent complexes to ERG target sites facilitating its chromatin occupancy and driving the transcriptional outcomes mediating transformation [71].

### 1.5. The MYC/AR axis

Alongside the disruptive potential of ERG, MYC overexpression represents another critical node in PCa epigenetic dysregulation. AR-driven MYC overexpression occurs in approximately 8% of primary tumors and 37% of metastatic disease, correlating with poor clinical outcomes [10,77]. Genome-wide studies mapping MYC and AR binding sites revealed 25–30% overlap between the sites, suggesting a MYC/AR signaling axis that distorts normal transcriptional programs [78]. At co-localization sites, AR exhibits higher binding affinity and enrichment for ERG, FOXA1, and HOXB13 motifs [78]. These MYC/AR regions are marked by enhancer-associated histone modifications, suggesting a role for 3D chromatin looping interactions in mediating their transcriptional effects [78]. Recent work has identified that these oncogenic enhanceosomes are co-occupied by AR/MYC acetylated histone modifications, which further stabilize the transcription factor complexes [79].

In mCRPC and NEPC progression, MYC overexpression antagonizes AR signaling through epigenetically modulated dedifferentiation and lineage switching. Using *in vivo*, *in vitro*, and *ex vivo* models, Berger etal. demonstrated that MYC overexpression is associated with diversification of the MYC cistrome, development of poorly differentiated foci, loss of epithelial markers (AR, KRT5/8) and gain of epithelial-to-mesenchymal transition and neuroendocrine markers (VIM, NCAM1) [10]. MYC enrichment at neural lineage genes correlates with decreased androgen responsiveness and increased bivalency at neural lineage genes, mediated by EZH2 [10].

Super-enhancers are large domains of densely clustered enhancer elements bound by master TFs that regulate cell-identity genes [12]. Both AR and MYC have been investigated in this context, as they preferentially bind within cell-type-specific enhancers, which frequently function as super-enhancers in regulating oncogenes [80]. Recent work by Guo etal. revealed that the MYC/AR axis converges at a developmental super-enhancer in CRPC [28]. They showed that AR represses MYC through redistribution of cofactors, including BRD4, where BRD4 and H3K27ac increase at AR binding sites upon androgen stimulation, and they concordantly decrease at MYC binding sites. At the prostate-specific MYC super-enhancer, androgen treatment perturbs the entire TAD, inhibiting MYC promoter – super-enhancer interactions while simultaneously increasing AR, FOXA1, HOXB13, and BRD4 binding at the super-enhancer, limiting MYC promoter contact [28]. These findings reveal an inverse interplay wherein MYC downregulation results from androgen-induced redistribution of its coactivators. MYC overexpression has also been shown to antagonize AR-dependent transcription by preventing pause-release of RNA Polymerase II at target gene promoters, resulting in nonproductive transcription [81]. Additionally, the chromatin remodeler BRG1 regulates MYC expression by mediating chromatin accessibility, with BRG1 knockdown establishing a suppressive chromatin state at the MYC promoter [82].

### 1.6. Therapeutic implications: targeting the chromatin landscape in prostate cancer

Understanding this complex, 3D-organized transcription factor network provides a map for new therapeutic interventions in prostate cancer (Figure 1). Transcription factors have historically been considered “undruggable” due to their intrinsically disordered interaction networks with functional partners. However, recent studies have challenged this dogma, demonstrating successful targeting of transcription factors in prostate cancer. Chemical inhibitors targeting AR ligand-binding domains, such as enzalutamide, represent the primary treatment for early-stage disease. Beyond their direct transcriptional effects, AR inhibitors also induce broader chromatin state changes that have immunological consequences. Recent studies suggests that AR inhibition leads to derepression of endogenous retrovirus (ERVs) [83] and upregulation of MHC-I expression [84]. This may be driven by altered chromatin accessibility landscape at repetitive elements that are normally silenced by AR-dependent epigenetic programs. Notably, potent AR stimulation can similarly induce ERV transcript expression and enhance PCa cell immunogenicity [85]. These findings suggest the possibility that AR-targeted therapies may be most effective when combined with immunotherapy, by leveraging their capacity to remodel the chromatin landscape at repetitive elements. Yet these agents become ineffective in castration-resistant states as tumors circumvent androgen-targeted therapies through AR amplification, point mutations, splice variants, and gene fusions.

Unlike genetic alterations, which remain challenging to correct, dysregulated epigenetic mechanisms are therapeutically tractable [86]. Currently, only a small number of epigenetic drugs have received FDA approval, including inhibitors of EZH2 (EZH2i), histone deacetylases (HDACi), chromatin remodelers (SWI/SNFi), and DNA methyltransferases (DNMTi) (Figure 1), with many additional candidates in clinical trials for solid tumors.

### 1.7. DNA methylation

While DNA methylation – the addition of methyl groups to cytosine bases at CpG dinucleotides by enzymes, such as DNMTs, has been the primary focus of epigenetic therapy development, its role in prostate cancer is highly context-dependent. In CRPC, adenocarcinoma and NE-like tumors exhibit distinct DNA methylation profiles, highlighting roles of DNA methylation in treatment resistance and AR-independent escape mechanisms [6]. DNA methylation instability in cancer associates with mutations in chromatin remodeling factors, supporting combined DNA methylation and chromatin remodeling inhibition as a therapeutic strategy [87]. In 22% of mCRPC tumors, Zhao etal. identified cancer-associated mutations in DNMT3B and the DNA demethylating enzyme TET2 [88]. In this cohort of 100 mCRPC samples, DNA methylation associated with expression of known PCa driver genes AR, MYC, and ERG, occurring preferentially at mutation hotspots and cis-regulatory regions [88]. Moreover, key androgen-response genes, such as *KLK3*, *NKX3-1*, and *FOLH1*, are correlated with increased promoter DNA methylation in mCRPCs compared to benign prostate samples [88].

Critically, “sentinel sites” where AR is reprogrammed during PCa and mCRPC progression exhibit hypomethylation compared to normal prostate tissue, with dysregulation of these sites linked to NE-like lineage switching [43]. In treatment-induced NEPC, newly gained FOXA1 binding sites and enhancers similarly show hypomethylation compared to normal and primary disease [39]. Furthermore, FOXA1 chromatin binding is promoted by LSD1-mediated histone demethylation in prostate cancer, illustrating the interplay between histone modification and DNA methylation in regulating pioneer factor activity [39].

While preclinical evidence supports DNA methylation inhibitors as a viable therapeutic strategy for androgen-driven PCa, translating this to clinical practice requires a deeper mechanistic understanding of how DNA methylation integrates with other layers of epigenome. Importantly, DNA methylation does not operate as an isolated linear mark but is functionally embedded within the 3D chromatin landscape, where DNA hypermethylation at enhancers can directly constrain enhancer-promoter loop formation [17,89] and can be targeted with DNMT inhibition [20,90], suggesting an active or consolidating role of DNA methylation in the reorganization of 3D chromatin topology.

### 1.8. ATP-Dependent chromatin remodelling

SWI/SNF complex subunits have emerged as particularly promising epigenetic therapy targets, especially for NEPC. These complexes play critical roles in chromatin remodeling and are mutated in over 20% of cancers [91]. BRM (SMARCA2) and BRG1 (SMARCA4) are the most extensively studied subunits and are highly overexpressed in aggressive PCa states. These subunits engage in direct interactions with lineage-specific factors in both CRPC [92] and NEPC-specific contexts [93].

A study by Cyrta etal. revealed pro-tumorigenic SWI/SNF alterations in CRPC and NE PCa that result in disease-stage-specific cofactor binding [93]. These cofactors include NKX2.1, CHD4, MTA1, and VGF uniquely in NE PCa, and the AR-associated pioneer factor HOXB13 specifically in CRPC [93]. The NE-specific cofactors are particularly intriguing given their known roles in coordinating neural-like lineage plasticity that drives subtype switching [93].

Dual BRG1/BRM inhibition has shown remarkable preclinical promise, partially due to its ability to induce large-scale chromatin remodeling, rather than targeting individual transcription factors directly. Xiao etal. demonstrated that PCa cells with AR and FOXA1 positivity exhibit high sensitivity to AU-15330, a proteolysis-targeting chimera (PROTAC) that degrades SMARCA2 and SMARCA4 [94]. Degradation triggers immediate dissociation of AR, FOXA1, MYC, and ERG from enhancers as chromatin accessibility reduces at their binding sites, reflecting dependence of these oncogenic transcription factors on SWI/SNF-mediated nucleosome remodeling to maintain accessible chromatin at their target regulatory elements. These chromatin alterations produce direct transcriptional consequences, with loss of enhancer-promoter loops resulting in significant downregulation of oncogenes regulated by AR, FOXA1, MYC, and ERG. AU-15330 inhibited PDX tumor growth and showed synergy with enzalutamide, achieving regression in approximately 30% of cases with minimal toxicity [94], further supporting the therapeutic relevance of targeting chromatin topology as a unifying vulnerability across multiple oncogenic transcription factor networks.

However, the translation of SWI/SNF targeting into the clinic faces important challenges, including the potential for off-target toxicity given the broad role of SWI/SNF complexes in normal tissue homeostasis, the risk of resistance through compensatory chromatin remodeling mechanisms, and the need to identify predictive biomarkers that can prospectively select patients most likely to benefit from this approach.

### 1.9. Histone modifications

Histone deacetylases (HDACs) are enzymes that remove acetyl groups from histone proteins in chromatin, which can lead to a tighter packing of DNA and a reduction in gene expression. HDACs are frequently overexpressed in prostate cancer and play multifaceted roles in AR signaling. AR-mediated transcription involves recruitment of AR and coactivators that have histone acetyltransferase activity to AR target gene promoters, leading to acetylation and active transcription [30,31,95]. On the other hand, HDAC1, HDAC2, and HDAC3 are enriched at ERG-AR interaction hubs where they mediate transcriptional repression [29]. Consistently, HDAC inhibitors function by reducing androgen receptor activity [96,97]. HDAC inhibition can therefore resensitize castration-resistant cells to androgen deprivation by destabilizing AR, disrupting AR-coactivator complexes, and disrupting the precise chromatin acetylation patterns required for AR-mediated transcription [98]. Further, in the mCRPC setting, profiling of these H3K27ac decorated chromatin landscapes has been used to predict resistance to AR inhibition and identify signatures of sensitivity to HDAC inhibitors [99].

Importantly, the effects of HDAC inhibition may extend beyond local histone modification changes to alter 3D genome organization. Recent work using stem cell models demonstrated that transient HDAC inhibition can induce lasting changes in 3D genome folding, with perturbed chromatin topology partially maintained even after withdrawal of the initial trigger [100]. This suggests a possibility that HDAC inhibition in CRPC may exert durable effects on AR-driven transcriptional programs not only through acute disruption of histone acetylation but by persistently remodeling the 3D enhancer-promoter connectivity.

Despite mechanistic rationale, clinical trials with pan-HDAC inhibitors have shown limited single-agent activity in CRPC, with modest response rates and dose-limiting toxicities. Several factors likely underlie this gap between preclinical results and clinical performance: (1) pan-HDAC inhibitors lack isoform selectivity, resulting in broad disruption of normal tissue acetylation and a narrow therapeutic window; (2) compensatory activation of parallel chromatin remodeling pathways can maintain AR-driven transcriptional output despite HDAC inhibition; and (3) clinical trials have largely been conducted without biomarker stratification to identify patients most likely to benefit. Suberoylanilide hydroxamic acid (vorinostat) has demonstrated ability to downregulate AR expression and inhibit proliferation in preclinical models [101]. Similarly, HDAC6 inhibitors (ricolinostat, citarinostat) target cytoplasmic HDAC6, which stabilizes AR by deacetylating HSP90, showing improved tolerability profiles [102,103]. HDACi plus AR-targeted therapy has shown synergistic growth inhibition in preclinical models and is being evaluated in clinical trials with encouraging preliminary results [101,104]. However, the lack of specificity and off-target effects have limited their clinical applications [105]. Isoform-selective approaches combined with H3K27ac-based patient stratification could represent the most promising path to realizing the clinical potential of this drug class in CRPC.

EZH2 inhibitors represent another important class targeting histone methylation. EZH2, a polycomb group protein, was first identified as a key driver of mCRPC in 2002 by the Chinnaiyan lab, with overexpression linked to aggressive disease and poor prognosis [106]. Epigenetic dysregulation involving EZH2 contributes to prostate cancer lineage plasticity and neuroendocrine differentiation [11]. While several EZH2 inhibitors (e.g., CPI-1205 [107], tazemetostat [108], SHR2554, tulmimetostat [109]) have failed to show clinical benefit in prostate cancer, recent data on mevrometostat are promising. In a phase 1/2 trial, mevrometostat plus enzalutamide significantly improved radiographic progression-free survival (rPFS), objective response rate, and PSA50 response compared to enzalutamide alone, with manageable gastrointestinal toxicity mitigated by dosing with food [110]. Two ongoing phase 3 trials (MEVPRO-1 and MEVPRO-2) are now evaluating mevrometostat in combination with enzalutamide in both pretreated and treatment-naïve mCRPC settings [110].

While histone deacetylase inhibitors and EZH2 inhibitors have entered clinical development, their efficacy as monotherapies has been modest. This likely reflects the redundancy and compensatory mechanisms within epigenetic regulatory networks. This has also been observed in relation to histone acetyltransferases, where dual p300/CBP degradation is able to overcome compensatory mechanisms that limit single-target approaches [79]. Future therapeutic strategies may require rational combinations targeting multiple nodes of chromatin regulation, for example, combining EZH2 inhibitors with SWI/SNF degraders to simultaneously disrupt histone methylation patterns and chromatin accessibility.

The optimal sequencing of these agents is likely to be context-dependent, informed by the epigenetic state of the tumor at each stage of disease progression. For instance, using EZH2 inhibitors such as mevrometostat early to reverse polycomb-mediated silencing, as a priming agent, before introducing SWI/SNF degraders to prevent chromatin remodeling-driven escape [111]. Preclinical evidence increasingly supports the concept that sequential rather than concurrent targeting can maximize therapeutic vulnerability by exploiting the transient epigenetic states created by the first agent [112]. Time-resolved epigenome profiling will be essential to define these windows of opportunity to realize the full clinical potential of combinatorial epigenetic therapy in prostate cancer.

### 1.10. Future perspectives

**Figure 2.**
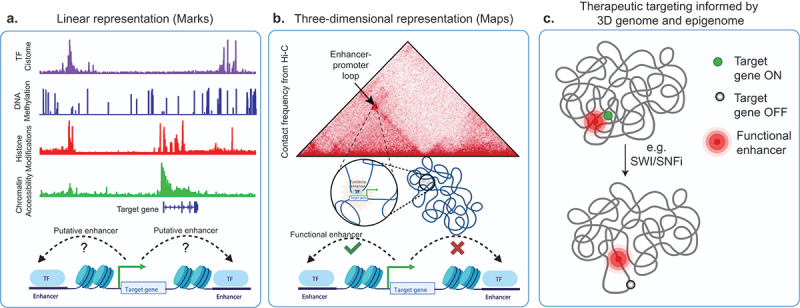
Linear Marks versus 3D Maps. Interpreting epigenetic remodeling by predicting enhancer-promoter interactions using linear representation (Marks) versus three-dimensional representation (Maps). (a) In the context of linear genome multiple TF-bound putative enhancer elements enriched for TF binding, DNA hypomethylation, active histone modifications and chromatin accessibility are predicted to module the expression of the target gene based on their linear proximity. (b) In the context of the 3D genome, spatial proximity based on enhancer-promoter looping interactions detected by Hi-C informs the “functional” enhancer element (or elements) for the target gene. (c) Epigenetic inhibitors, such as SWI/SNFi, can disrupt oncogenic 3D chromatin interactions between target gene (green circle) and functional enhancer (red circle) by directly targeting 3D chromatin structure and dynamics, resulting in the target gene being switched off. Created in BioRender. Achinger-Kawecka, J. (2026) https://BioRender.com/am523pk.

Abbreviations: TF: Transcription factor, SWI/SNFi: Switch/Sucrose Non-Fermentable (SWI/SNF) complex inhibitors.

The primary challenge in treating androgen-driven prostate cancer lies in translating complex epigenetic dysregulation into actionable therapeutic targets. Recent evidence suggests that analyzing a single layer of epigenomic information through linear “marks” provides an incomplete view of the chromatin landscape. To achieve precise patient stratification, we must shift from a linear “single-modality” interpretation of epigenetic marks to comprehensive multi-omics characterization 3D “maps” (Figure 2).

A robust 3D epigenome map should integrate the input from the linear epigenetic marks with the functional chromatin interactions driving gene expression in a normal cell or aberrant gene expression in a cancer cell (Figure 2(a)): 1) Cistrome Profiling: To map transcription factor binding and identify disease-specific enhancer usage; 2) DNA Methylation Mapping: To capture stably silenced regulatory regions and enhancer reprogramming, 3) Histone Modification Landscapes: To distinguish between active, poised, and repressed chromatin states; 4) Chromatin Accessibility Mapping: To define open chromatin regions and predict transcription factor occupancy; and 5) 3D Chromatin Architecture: To identify functional long-range enhancer-promoter interactions at target genes (Figure 2(b)). By integrating these five different epigenetic layers in prostate cancer, we can pinpoint specific epigenetic features at regulatory regions that dictate drug sensitivity for each patient. For instance, tumors driven by increased chromatin contacts with super-enhancers may be vulnerable to SWI/SNF inhibitors, as recently demonstrated in the context of CRPC [94] (Figure 2(c)).

A key limitation is that much of the mechanistic evidence underpinning this framework is derived from preclinical models and in vitro systems, and that translating multi-layered 3D epigenome profiling into routine clinical practice presents a real technical and logistical challenge. While recent technological advances have now substantially improved the adaptation of these techniques to limited tumor material available from clinical biopsies [113], prospective clinical validation in cohorts with linked treatment outcomes and standardized bioinformatic pipelines will be essential to realize the translational potential of this integrative epigenomic framework.

The remarkable plasticity of prostate cancer, particularly its transition to neuroendocrine prostate cancer (NEPC), represents both a clinical challenge and a significant opportunity [6,114]. Rather than viewing lineage switching solely as an evolutionary escape mechanism to be avoided, we should consider how this plasticity might be exploited therapeutically. By mapping the chromatin states that govern these transitions, we may be able to direct tumor differentiation toward therapeutically favorable phenotypes or, conversely, lock tumors into states that remain vulnerable to standard-of-care treatments.

Pioneer factors such as FOXA1 offer a potential focus in this context. While complete inhibition of FOXA1 is likely precluded by its essential role in normal tissue homeostasis, the ability to modulate its activity or redirect its binding could effectively reshape available lineage trajectories. Simultaneously, preventing the chromatin remodeling necessary for lineage plasticity, for example, through SWI/SNF inhibition, might lock tumors in states where standard-of-care therapies, such as androgen inhibition retain efficacy and prevent lineage switching. The emerging success of PROTAC degraders targeting SWI/SNF subunits opens a new treatment avenue: by selectively degrading rather than inhibiting the enzymatic activity of epigenetic regulators, we gain the ability to disrupt non-enzymatic scaffolding functions while potentially achieving greater selectivity.

Future therapeutic development should prioritize the design of degraders or inhibitors tailored to specific components of the androgen receptor transcriptional network, for example selective degradation of ERG in fusion-positive tumors or the targeted removal of specific co-factors that drive treatment resistance. Beyond inhibition and degradation, the development of modulators designed to disrupt protein-protein interactions within transcription factor complexes represents a highly promising approach, offering a potential for greater specificity.

Despite this potential, several barriers prevent the immediate clinical application of epigenetic remodeling including:

1. Redundancy and Efficacy: Single-agent epigenetic therapies often show modest results because of the inherent robustness and redundancy in the epigenetic networks.
2. Toxicity vs. Synergy: While combinations of epigenetic drugs show promise in preclinical models, early clinical trials have been hampered by high toxicity.
3. Optimal Sequencing: Success may depend on “epigenetic priming” - using epigenetic therapies to reset the chromatin landscape before administering immunotherapies or utilizing chronic maintenance to prevent epigenetic evolution.

Finally, the promise of targeting 3D epigenome remodeling extends beyond prostate cancer to other malignancies driven by transcription factor networks and epigenetic plasticity. Further understanding of the interplay between the 3D genome and epigenome and transcription factor networks will help to create more selective therapies and optimize the efficacy of epigenome-based therapies.

## Article highlights

- **Central Role of 3D Chromatin Architecture**: Prostate cancer progression and treatment resistance are driven by the dynamic 3D reorganization of the epigenome, which facilitates aberrant transcription factor networks beyond linear DNA modifications.
- **AR-Driven Loop Dynamics**: Androgen receptor (AR) activity is characterized by rapid and transient changes in 3D chromatin loops, where specific “dominant enhancer” loops within multi-enhancer networks disproportionately drive oncogenic gene expression.
- **Pioneer Factors as Architectural Master Regulators**: Key transcription factors like FOXA1, HOXB13, MYC and ERG act as architectural nodes, coordinating chromatin accessibility and structural variation (including ecDNA-mediated loops) to sustain the AR cistrome during disease evolution.
- **Epigenetic Lineage Plasticity**: The transition to aggressive states, such as neuroendocrine prostate cancer (NEPC), involves large-scale 3D chromatin reprogramming and loss of compartmentalization, which provides a unique window for therapeutic intervention.
- **Targeting Chromatin Topology**: Next-generation epigenetic therapies, including SWI/SNF degraders, EZH2 inhibitors and isoform-selective HDAC inhibitors, offer a promising strategy to disrupt the physical 3D interactions that support oncogenic transcriptional programs.
- **Integrated 3D Epigenomic Mapping**: Future precision medicine in prostate cancer requires a shift from linear single-layer epigenetic profiling to comprehensive 3D multi-omics maps that integrate TF cistromes, DNA methylation, and 3D chromatin structure and dynamics to identify patient-specific vulnerabilities.
